# Case report: Bilateral damage to the immature optic radiation and secondary massive loss of retinal ganglion cells causing tunnel vision

**DOI:** 10.3389/fnins.2023.1143044

**Published:** 2023-04-04

**Authors:** Maria Nilsson, Finn Lennartsson, Hanna Maria Öhnell, Lotta Gränse, Lena Jacobson

**Affiliations:** ^1^Unit of Optometry, Department of Clinical Neuroscience, Karolinska Institutet, Stockholm, Sweden; ^2^Diagnostic Radiology, Department of Clinical Sciences Lund, Lund University, Skåne University Hospital, Lund, Sweden; ^3^Ophthalmology, Department of Clinical Sciences Lund, Lund University, Skåne University Hospital, Lund, Sweden; ^4^Section for Eye and Vision, Department of Clinical Neuroscience, Karolinska Institutet, Stockholm, Sweden

**Keywords:** cerebral visual impairment, white matter damage of immaturity, retrograde trans-synaptic degeneration, tunnel vision, optical coherence tomography

## Abstract

We describe the case of a 30-year-old woman, who needed a formal report on her visual impairment to seek support from society. She was born preterm, and during her neonatal period, she suffered from bilateral intraventricular hemorrhage (IVH) grade 3, a condition that can cause cerebral visual impairment (CVI) due to damage to the retro-geniculate visual pathways. Individuals with such brain damage of this severity are often restricted by cerebral palsy (CP) and intellectual disability, and thus have a limited ability to cooperate in the assessment of visual function. However, our patient was capable of providing reliable test results, and she manifested only a small island of central vision in each eye, with additional reduced visual acuities. She cooperated well in examinations involving MRI of the brain, optical coherence tomography (OCT) of retinal ganglion cells, and multi-focal visual evoked potentials, with each test providing information about potential limitations in the structural prerequisites for visual function. What distinguishes our case is the severity of the damage to the optic radiations and the massive secondary loss of most of her retinal ganglion cells (GCs). However, there is some measurable visual function, which may be due to developmental neuroplasticity during early development, when surviving GCs prioritize the central visual field. Despite her visual difficulties, she is a keen portrait painter. Our patient may be representative of, and a spokesperson for, other individuals with extensive brain damage of the same etiology, who are unable to perform perimetric tests and therefore run the risk of not being recognized as severely visually impaired, and consequently, not being given the best conditions for habilitation. OCT may serve as a helpful diagnostic tool.

Aim: This study aims to describe visual behavior and practical applications of visual function in relation to structural prerequisites for visual function.

## Introduction

The number of children identified with cerebral visual impairment (CVI) is increasing because of higher rates of survival among critically ill infants (Mitry et al., 2013). CVI in children displays a range of visual deficits. Visual function is characterized by reduced input, which is further complicated by perceptual cognitive dysfunction. Subnormal linear visual acuity (VA) and restriction, or subnormal sensitivity, of the visual field (VF) function are commonly seen, often in combination with ocular motility problems such as nystagmus, strabismus, inadequate saccades and smooth pursuit movements. Children suffering from CVI often exhibit other symptoms of cerebral dysfunctions including sensorimotor impairment ranging from mild motor problems to cerebral palsy (CP) (Chokron and Dutton, 2016), as well as a wide range of learning disabilities (Chokron et al., 2021).

Damage to the immature optic radiation is a common cause of CVI in prematurely born individuals (Teoh et al., 2021). The etiology of such damage is often perinatal hypoxic ischemia causing either intraventricular hemorrhage (IVH; Volpe, 1989), which can be potentially complicated by post-hemorrhagic ventricular dilation or periventricular hemorrhagic infarction, or periventricular leukomalacia (PVL; Shuman and Selednik, 1980); however, IVH and PVL can occur together (Kusters et al., 2009). Lesions occurring at gestational age 24–34 weeks are commonly referred to as white-matter damage of immaturity (WMDI) (Bax et al., 2006), as they can occur in the premature neonate or *in utero*. IVH and PVL are the dominating lesions types of WMDI and have a predilection for the peritrigonal areas and the upper parts of the optic radiations (Shuman and Selednik, 1980) also seen on imaging (Rutherford et al., 2010).

Retrograde trans-synaptic degeneration (RTSD) from the lesion across the geniculate body leads to the degeneration of retinal ganglion cells (Jindahra et al., 2009; Dinkin, 2017). The pattern of ganglion cell (GC) loss may predict VF outcome. With tools such as MRI tractography and optical coherence tomography (OCT), we have been able to illustrate such association (Lennartsson et al., 2014, 2018). VF-deficits in children with WMDI have been described as unilateral or bilateral homonymous defects most often affecting the inferior VFs (Dutton et al., 2004; Jacobson et al., 2006). In individuals with bilateral WMDI, we have noticed relative sparing of the very central VF, possibly due to the plasticity of the immature visual system (Lennartsson et al., 2021). This case illustrates that visual input may be severely restricted by bilateral damage of the optic radiation with RTSD of a majority of the retinal GCs, and yet there is measurable, but subnormal VA in combination with tunnel vision.

## Aim

To describe visual behavior and practical applications of visual function in relation to structural prerequisites for visual function.

## Case description

We report on the findings of a woman born at 30 weeks of gestation with an emergency cesarean due to placental ablation. Birth weight was 1,654 g. During transportation to the neonatal intensive care unit in another hospital, she had an episode of severe asphyxia. During the second day of post-partum, she suffered from bilateral ultrasound-verified IVH grade 3. Ventricular sizes increased initially, then stabilized, and shunt surgery was not necessary. No retinopathy of prematurity was detected. She was discharged from the hospital after 75 days.

As an infant, our patient had a psycho-motor developmental delay. Deconjugated eye movements were noted at 2 months corrected age. At 1 year old, she was diagnosed with esotropia of the left eye and mild bilateral spastic CP, left more than right. She had intense physiotherapy ad modum Vojta (Vojta, 1981; De-La-Barrera-Aranda et al., 2021) and was able to walk without support at the age of 3½ years. To avoid strabismic amblyopia, the ophthalmologist prescribed patching of the right eye for 2–3 h per day from the age of 1 year after determining the optic disks and maculae as normal through indirect ophthalmoscopy. At the age of 2 years, she was prescribed one drop of 0.5% atropine a day in the right eye, which was carried through for 3 months. During this treatment, she still had esotropia in her left eye, and therefore partial patching was prescribed until she was 4½ years. At the age of 5, it became obvious that our patient was visually impaired, with subnormal best corrected visual acuity (BCVA) in both eyes, esotropia, and problems to fixate and follow. VF function was assessed with confrontation. She had problems maintaining fixation, and she did not respond to peripheral stimuli until it was near the fixational line. At the age of 6, a more extensive workup was performed including an ophthalmological examination and assessment of vision, and she was diagnosed with CVI. It was assumed that she was unable to follow the instructions because severely restricted VFs are uncommon in children with CVI.

At the age of 22 years, our patient was cognitively assessed with Wechsler Adult Intelligence Scale-IV. Her intellectual profile was uneven, with the best performance in verbal comprehension tasks (index score 69) and working term memory tasks (index score 62), while she obtained lower scores in tasks involving perceptual reasoning function (index score 50) and visual processing speed (index score 50). The total IQ was 52.

Abstract reasoning was difficult. She struggled with math and could not handle money. She also had a low perception of time. She could read print with magnification and a generous amount of time, and she enjoyed “easy to read”-books. She required more time to complete visual tasks and had difficulties in drawing conclusions from the visually presented material. The color was helpful to recognize objects in pictures, whereas geometrical shapes were difficult to identify. Spatial orientation was extremely difficult for her. She could find her way around her own house and the closest surroundings, but she could not find her way outside of this very familiar area. She could not estimate direction, depth, or distance, e.g., if a car was approaching.

## Results

### Visual function and retinal structure

**Figure 1:**
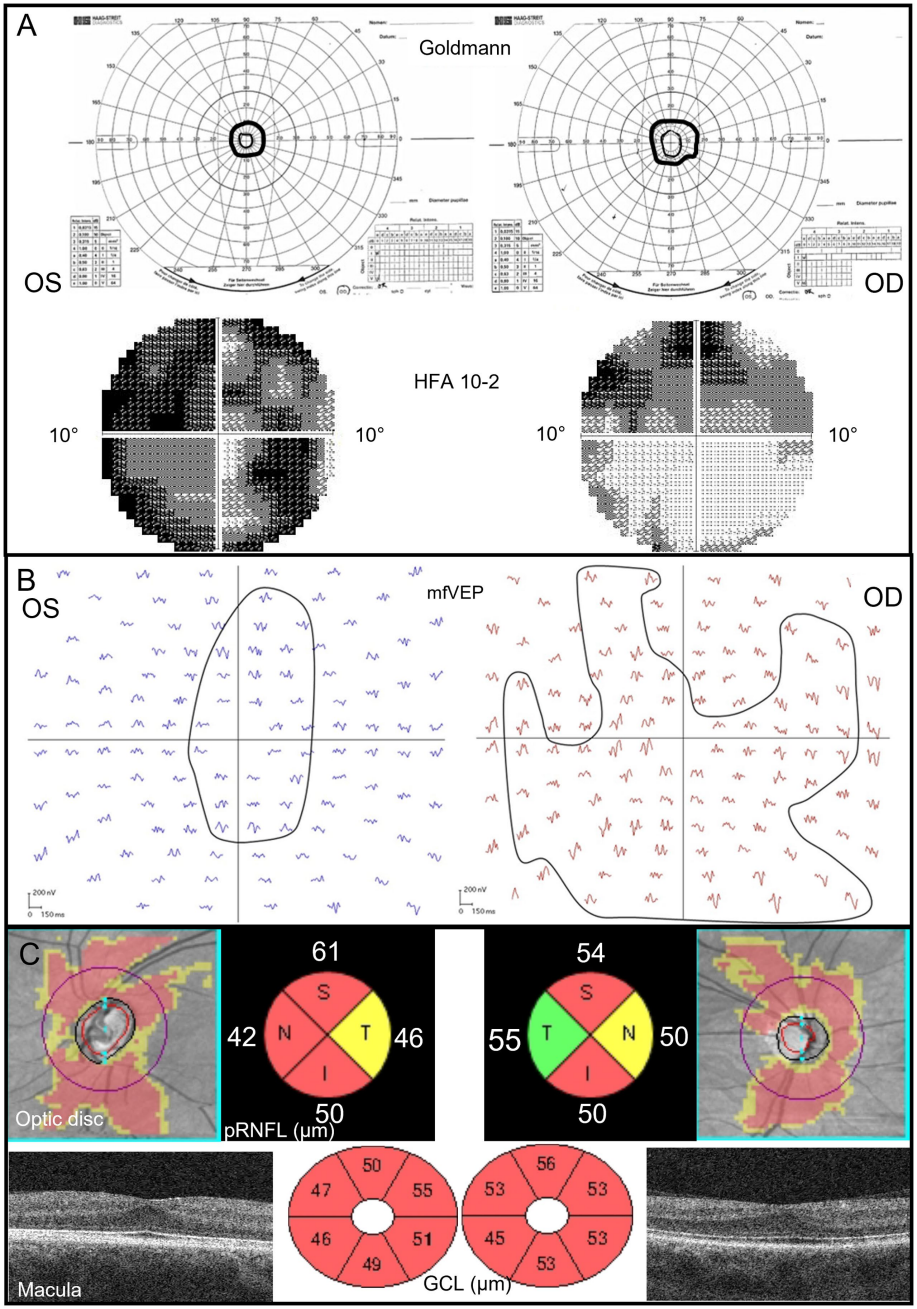
**(A)** Goldmann perimetry shows completely constricted visual fields (tunnel vision). HFA 10–2 shows a marked reduction, especially in the left eye, with the best function in the lower parts of the central field of the right eye. **(B)** MfVEP responses from the left and right eye describe the cortical response correlating to the central 20–25-degree VF. Amplitudes with the best signal-to-noise ratio are more concentrically constricted in the left eye, and the amplitudes are generally lower in this eye than the amplitudes from the right eye. **(C)** OCT images are color-coded according to red = outside normal limits, yellow = borderline, and green = within the limits of the reference database. All retinal thickness values are in μm. The pRNFL topography shows a generally thin peripapillary nerve fiber layer with some sparing in the temporal quadrants in the right eye. The macular GCL topography indicates a very thin layer in both eyes and is most pronounced in the left eye. The foveal pit is shallow in both eyes and shows signs of inhibited migration of the inner retinal layers centrally. OS, left eye; OD, right eye; S, superior; N, nasal; I, inferior; T, temporal; pRNFL, peripapillary retinal nerve fiber layer; GCL, ganglion cell + inner plexiform layer; and HFA, humphrey field analyzer.

Our patient asked for the assessment of visual function at 30 years of age. Under binocular conditions, her best BCVA (Snellen) was 20/80 in the right eye (−2.0 = −2.5 × 120°) and 20/200 in the left eye (+0.75 = −1.25 × 45°) at distance and 20/60 at near. She had exotropia of the left eye and nystagmus, as well as difficulties in maintaining stable fixation. Contrast sensitivity was estimated with a MARS chart and classified as moderate to severely reduced (log CS 1.6). During the color sensitivity test, Farnsworth D-15 test, there were several mistakes along the deutan and protan axes in both the right and left eye, indicating a severe defect in one of these axes. Goldmann perimetry demonstrated tunnel vision restricted to 15 degrees in the right eye and 10 degrees in the left eye, and computerized perimetry with Humphrey Field Analyzer (HFA) showed reduced sensitivity within the small central fields (Figure 1A). The mean deviation in the HFA 10–2 fields was −15.53 dB in the right eye and − 25.32 dB in the left eye. Multi-focal visual evoked potential (mfVEP) reflects the cortical activity topographically corresponding to the central 25-degree VF. Localized lesions in the central visual field can, therefore, consequently be identified (Hood et al., 2003). In our patient, mfVEP (VERIS-system) showed cortical response corresponding to the central 25-degree VF from the right eye with a scotoma in the upper part. The amplitudes with the best signal-to-noise ratio correlated with the central 20–25 degrees VF in the lower part of the VF (Figure 1B). In the cortical responses from the left eye, the amplitudes with the best signal-to-noise ratio were corresponding to approximately 20 degrees in the superior VF, approximately 7 degrees in the inferior VF, approximately 1.5 degrees in the temporal VF, and approximately 3 degrees in the nasal VF. In the left eye response, there was a sharper decline in the periphery compared to the right eye (Figure 1B). Generally, the amplitudes in the response were higher from the right eye compared to the left eye. This matched well with better visual acuity in the right eye and the asymmetry in VF function between eyes. The peripapillary retinal nerve fiber layer (pRNFL) and ganglion cell + inner plexiform layer thickness (GCL) as measured with OCT (Zeiss Cirrus HD OCT 5000) showed to be markedly reduced in both eyes and most sectors at a level considered to be floor values for retinal thickness (Leite et al., 2012; Bowd et al., 2017; Figure 1C). In an eye with the normal distribution of axons, the pRNFL would be considerably thicker in the superior and inferior quadrants compared to the nasal and temporal. In this case, the thickness was reduced in the superior and inferior sectors and best preserved in the temporal quadrants, especially in the right eye. The temporal pRNFL, i.e., the peripapillary macular bundle, supplies the central VF. Therefore, it is not surprising that this portion of axons was relatively closest to normal when considering that only the central VF is preserved. At the same time, the ganglion cell layer in the macula was thin in both eyes and most pronounced in the left eye with the lowest function, indicating a strong structure–function relationship. In contrast, the optic disk in the right eye seemed smaller, showed larger cupping, and was paler compared to the left eye. None of the disks, based on fundus photos, appeared as excavated as could be expected based on function tests or OCT findings.

### MRI

**Figure 2:**
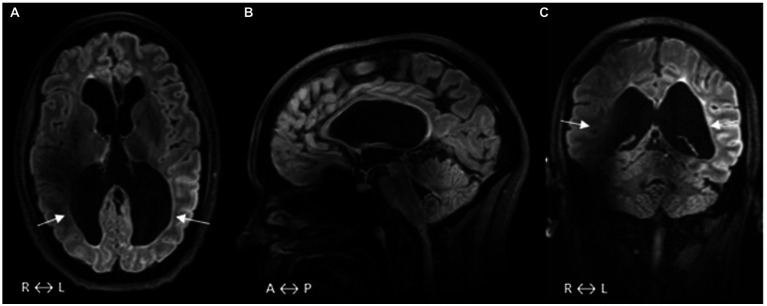
**(A–C)** FLAIR-MRI acquired at 7 T. **A–C** show a general loss of WM with secondary ventricular dilatation **(A)**, small areas of gliosis in the periventricular WM, and size reduction of thalami **(A)**. The WM volume loss is most pronounced in the parietal regions with the expected affection of each of the ORs (arrows **A** and **C**). Midsagittal slice **(B)** shows that the corpus callosum is extremely thin, especially its posterior part. Magnetic field inhomogeneities, a common problem at 7 T, result in signal inhomogeneity/loss in the vicinity of tissue/air interfaces such as temporal bones and paranasal sinuses **(A–C)**. WM, white matter; FLAIR, fluid-attenuated inversion recovery; OR, optic radiation; R, right; L, left; A, anterior; and P, posterior.

Neuroimaging had not been performed previously. At the age of 31, she took part in a research study in 2021/22 which included a brain MRI at 7 T. These images showed a moderate to severe reduction of the white matter, most pronounced in the parietal regions, periventricular gliosis, and secondary ventricular dilatation (Figure 2). Both thalami were reduced in size and the corpus callosum very thin. The findings are compliant with the chronic state of a high-grade intraventricular hemorrhage, where the small areas of periventricular gliosis can be an expression of focal non-cystic PVL. This affects the retro-geniculate visual pathways with destructive white matter injuries along the course of the optic radiations.

## Discussion

This case demonstrates that damage to the immature retro-geniculate visual pathways cause RTSD of the retinal GCs and axons that correlate with the VF loss. Our patient has no peripheral VF function, markedly reduced sensitivity in the central field, and subnormal visual acuity levels.

We find her unique in being able to participate in all the examinations despite severe visual impairment, CP, and intellectual deficits. Our patient is a spokesperson for other individuals with extensive bilateral WMDI who, due to even more severe multiple functional disabilities, cannot cooperate in neither assessment of visual function nor other demanding objective examinations. The severity of their visual impairment, therefore, often remains underestimated.

As a child, the poor result of the VF examination was interpreted as affected by the inability to understand the instructions rather than completely restricted VFs. VF examination requires a strong focus, and the ability to resist the impulse to look at the stimuli and is in general very demanding for someone with poor vision. When examining children at high risk of CVI, easy and objective examinations such as OCT, which can predict VF function, are important. OCT can give the ophthalmologist information about the prerequisites for visual development and VF function. MfVEP and MRI confirmed the result indicated by OCT; therefore, OCT can be used as a standalone examination in similar cases. In addition, this case illustrates how difficult it may be to detect severe axonal loss due to RTSD by the examination of the optic disks with photography or ophthalmoscopy.

Difficulties in diagnosing may cause inefficiency in habilitation and/or inappropriate treatment in relation to the visual prerequisites. For our patient, treatment with the occlusion and atropinization of the best eye was prescribed to escape strabismic amblyopia. Such treatment may have hampered visual development in the better eye. These individuals also run the risk of being incorrectly diagnosed and treated for glaucoma in adulthood due to cupping of the optic disk (Brodsky, 2001; Beltagi et al., 2022).

This case demonstrates that visual input, through the few surviving GCs left after RTSD caused by bilateral WMDI, may be minimal and the resulting severe visual impairment may resemble what we see in patients with advanced retinitis pigmentosa. The plasticity of the immature visual system may be responsible for using these “survivors” for function within the very central visual field thus using no visual power for the peripheral VF.

**Figure 3:**
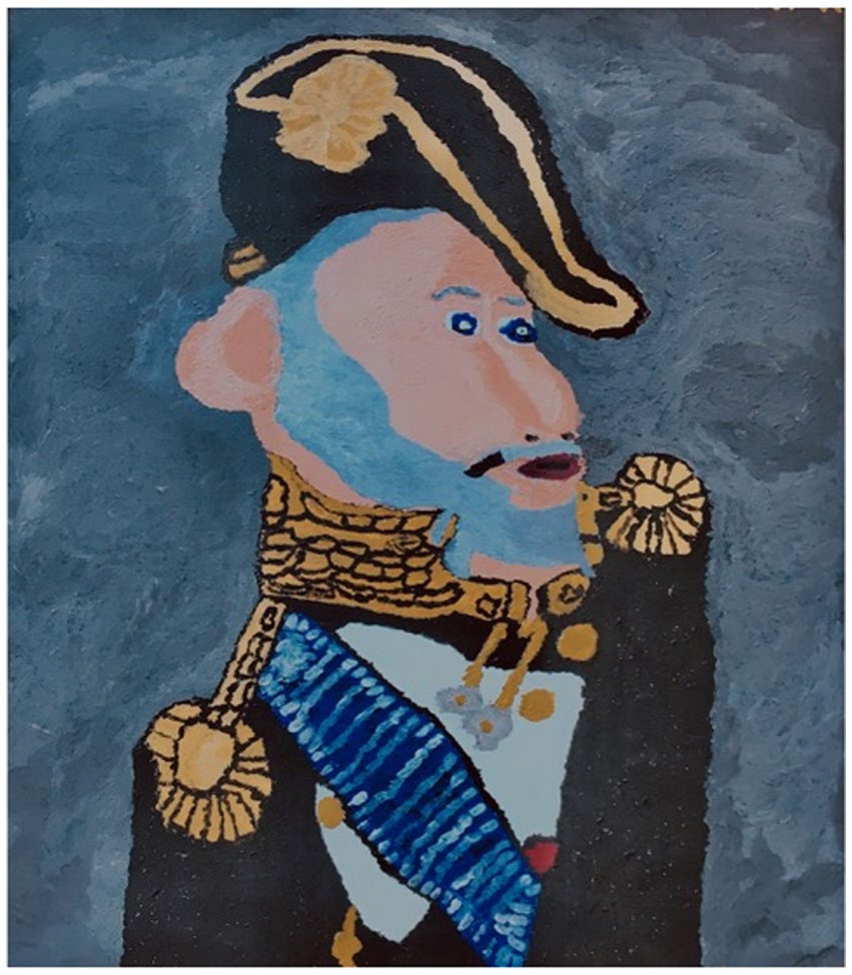
This artwork was painted by our patient in 2022, a painting inspired by an original portrait of former Swedish king Oscar II. This painting is an example of her practically applied vision.

In this case, we found a discrepancy between the structural measurements of vision, indicating a functional blind individual, and the practical visual function she exhibits. Despite severe visual impairment and reduced visual processing ability, our patient is today active as an artist, painting portraits with photos as models (Figure 3). One might find her ability to work with colors, shapes, and details remarkable in relation to her severe visual impairment.

## Data availability statement

The original contributions presented in the study are included in the article/supplementary material, further inquiries can be directed to the corresponding author.

## Ethics statement

The studies involving human participants were reviewed and approved by Regionala etikprövningsnämnden i Stockholm 2013/1114-31/2 and 2020-06677. The patients/participants provided their written informed consent to participate in this study.

## Author contributions

MN, FL, HÖ, LG, and LJ contributed to the conception and design of the study. FL performed an MRI examination. MN, LJ, HÖ, and LG performed the eye examinations. LJ gathered case background data and wrote the first draft of the background and case description. MN, FL, HÖ, and LG wrote the method and result sections. All authors contributed to the article and approved the submitted version.

## Funding

The study was financed by Swedish governmental funding of clinical research (ALF), Lions research fund Skåne, the foundation for the visually impaired in former Malmöhus län, the Crown Princess Margareta’s fund for visually impaired, and the Cronqvist’s foundation.

## Acknowledgments

The National 7T facility at Lund University Bioimaging Center is gratefully acknowledged for providing experimental resources.

## Conflict of interest

The authors declare that the research was conducted in the absence of any commercial or financial relationships that could be construed as a potential conflict of interest.

## Publisher’s note

All claims expressed in this article are solely those of the authors and do not necessarily represent those of their affiliated organizations, or those of the publisher, the editors and the reviewers. Any product that may be evaluated in this article, or claim that may be made by its manufacturer, is not guaranteed or endorsed by the publisher.
